# PP2A Inhibition Assay Using Recombinant Enzyme for Rapid Detection of Okadaic Acid and Its Analogs in Shellfish

**DOI:** 10.3390/toxins2010195

**Published:** 2010-01-25

**Authors:** Tsuyoshi Ikehara, Shihoko Imamura, Atsushi Yoshino, Takeshi Yasumoto

**Affiliations:** 1 Tropical Technology Center Ltd., 5-1 Suzaki, Uruma, Okinawa 904-2234, Japan; Email: shihoko@ttc.co.jp (S.I.); yoshino@ttc.co.jp (A.Y.); 2 Okinawa Science and Technology Promotion Center, 12-75 Suzaki, Uruma, Okinawa 904-2234, Japan; Email: yasumotot@subtropics.or.jp

**Keywords:** diarrhetic shellfish poisoning (DSP), okadaic acid (OA), protein phosphatase 2A (PP2A) inhibition assay, digestive gland (DG)

## Abstract

Okadaic acid and its analogs (OAs) responsible for diarrhetic shellfish poisoning (DSP) strongly inhibit protein phosphatase 2A (PP2A) and thus are quantifiable by measuring the extent of the enzyme inhibition. In this study, we evaluated the suitability of the catalytic subunit of recombinant human PP2A (rhPP2Ac) for use in a microplate OA assay. OA, dinophysistoxin-1(DTX1), and hydrolyzate of 7-*O*-palmitoyl-OA strongly inhibited rhPP2Ac activity with IC_50_ values of 0.095, 0.104, and 0.135 nM, respectively. The limits of detection and quantitation for OA in the digestive gland of scallops and mussels were 0.0348 μg/g and 0.0611 μg/g respectively, and, when converted to the whole meat basis, are well below the regulation level proposed by EU (0.16 μg/g whole meat). A good correlation with LC-MS data was demonstrated, the correlation coefficient being 0.996 with the regression slope of 1.097.

## 1. Introduction

Diarrhetic shellfish poisoning (DSP) refers to a gastrointestinal disease following the ingestion of bivalve shellfish containing dinoflagellate toxins of lipophilic nature collectively referred to as the okadaic acids (OAs): OA, dinophysistoxin-1 (DTX1 = 35-*R*-methyl OA), dinophysistoxin-2 (DTX2 = 31-demethyl-35-*S*-methyl OA), and their 7-*O*-acyl esters. The toxins accumulate mainly in the digestive gland (DG) and thus can be assessed by analyzing this tissue [1]. On top of the diarrheagenicity [2], the OAs possesses a tumor promoting activity, aggravating the potential health risks of DSP [3]. Concurrent with the OAs, other toxins called pectenotoxin (PTX), yessotoxin (YTX), and azaspiracids (AZA) often exist in the lipophilic fraction but are recommended not to be included in DSP based on the differences of the mode of actions [4]. As to the source of the toxins, a planktonic dinoflagellate *Dinophysis fortii* and 7 related species of *Dinophysis* were first shown to produce OA and DTX1 [5], followed by confirmation of DTX2 in *Dinophysis acuta*[6,7]. The benthic dinoflagellate *Prorocentrum lima* also produces OAs [8], though its involvement in DSP is arguable. The geographically wide distribution, frequent occurrence, and the important health risks that include potential tumor promotion have made DSP one of the top issues in seafood safety. The monitoring projects implemented in many countries not only test shellfish toxicity but also the toxic dinoflagellate species. Therefore, development of a good analytical method has been desired to implement monitoring efficiently and effectively.

OA is well known as a protein serine/threonine phosphatase inhibitor [9,10]. Protein serine/threonine phospahatses (PPs) are classified into four major classes: protein phosphatase (PP) 1, PP2A, PP2B, and PP2C [11]. OA inhibits these PPs to different extents: PP2A is inhibited most strongly, followed by PP1, and PP2B; PP2C is not inhibited at all [12]. Based on the specific inhibitory action, an assay method was proposed to determine OAs using *p*-nitrophenylphosphate (*p*NPP) as a substrate [13], and a colorimetric PP2A inhibition assay was developed [14,15]. Using native PP2A extracted from human red blood cells Tubaro *et al*. [15] revealed that the PP2A inhibition assay to be sensitive, accurate, reproducible, simple and rapid. However, the purity and stability of the enzyme available at the time did not meet the quality for use in kits. Thus, it is crucial to have a PP2A product of high purity and good stability to put a PP2A assay into practical use.

Recently, we produced the recombinant catalytic subunit of human PP2A (rhPP2Ac) by genetic engineering techniques using the baculovirus expression system with High Five insect cells [16]. The highly purified rhPP2Ac was biologically active and inhibited by natural toxins (OA and microcystin-LR). The evaluation of the PP2A inhibition assay using the rhPP2Ac for detecting and quantifying microcystins in environment water had been previously described [17]. In this study, we evaluated the rhPP2Ac for suitability to PP2A inhibition assay for OA detection in shellfish. 

## 2. Results

### 2.1. Comparison of the Inhibitory Effects of Lipophilic Toxins on rhPP2Ac Activity

**Figure 1:**
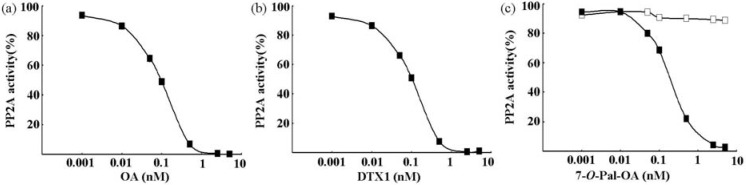
Inhibition of rhPP2A. The rhPP2Ac was assayed for *p*-NPP phosphatase activity in the presence of increasing concentrations of OA (**a**), DTX-1 (**b**), 7-*O*-Pal-OA (**c: open squares**), and hydrolyzate of 7-*O*-palmitoyl-OA (**c: closed squares**). Each point represents the mean (n = 3) with a standard error of less than 10%.

**Figure 2:**
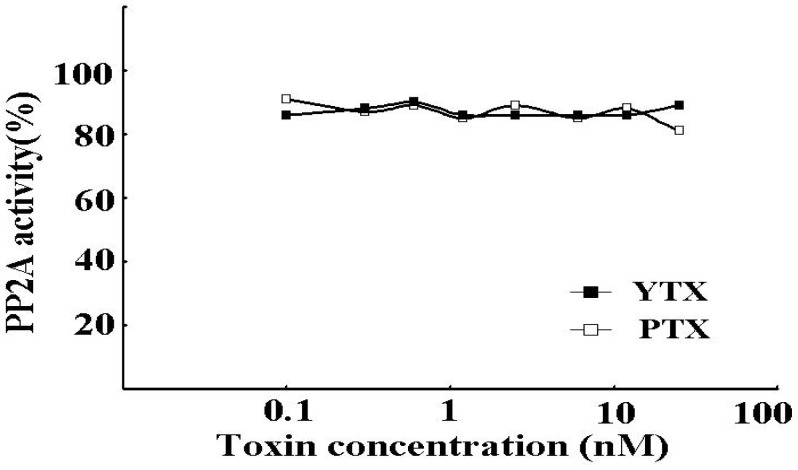
The inhibitory potency of YTX and PTX to rhPP2Ac. The rhPP2Ac was assayed for *p*-NPP phosphatase activity in the presence of increasing concentrations of YTX (closed squares) and PTX (open squares). Each point represents the mean (n = 3) with a standard error of less than 10%.

The inhibitory potency was examined by the PP2A assay. As shown in Figure 1, OA, DTX1, and pal-OA after hydrolysis but not before strongly inhibited the rhPP2Ac activity in dose-dependent manners at very low concentrations from 0.001 to 5 nM. On the other hand, PTX1 and YTX, the frequent concurrent toxins, did not inhibit rhPP2Ac at concentrations below 20 nM (Figure 2). Table 1 summarizes the IC_50_ values for the toxins tested: OA, 0.095 ± 0.007 nM; DTX1, 0.104 ± 0.006 nM; and hydrolyzate of 7-*O*-palmitoyl-OA, 0.135 ± 0.009 nM. These results indicate the specificity of the assay toward the OAs and the absence of interference from PTX and YTX.

**Table 1:** IC_50_ of lipophilic toxins in the PP2A inhibition assay.

**Toxins**	**IC**_50_(nM)
OA	0.095 ± 0.007
DTX	0.104 ± 0.006
7-*O*-Pal-OA	>10
Hydrolyzate of 7-*O*-Pal-OA	0.135 ± 0.009
TYX	> 20
PTX-1	> 20
All assays were performed in triplicate.	

### 2.2. Method Validation of the PP2A Assay

**Figure 3:**
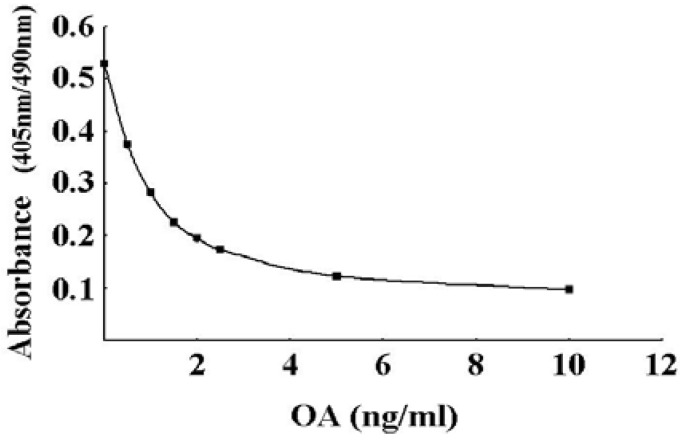
Calibration curve obtained by the PP2A assay. The PP2A assay was carried out with OA standard solutions (0, 0.5, 1.0, 1.5, 2.0, 2.5, 5.0, and 10.0 ng/mL). Each point represents the mean (n = 3) with SD of less than 1.5%.

To verify the suitability of rhPP2Ac for use in the routine monitoring of DSP toxins, we performed a validation study. First, the quantifiable range of the PP2A assay was assessed from the calibration curve prepared with known concentrations of OA. From the calibration curve shown in Figure 3, the quantifiable range was deduced to be in a range 0-10 ng/mL.

The limit of detection (LOD = the average of matrix blanks plus 3 SD) and the limit of quantitation (LOQ = the average of matrix blanks plus 10 SD) were calculated from 6 measurements of the unhydrolyzed and hydrolyzed matrix blanks prepared from digestive glands (DG) of mussels and scallops (Table 2). With blanks from mussels, the LOD values were 0.0424 μg/g (unhydrolyzed) and 0.0476 μg/g (hydrolyzed), and the LOQ values were 0.0725 μg/g (unhydrolyzed) and 0.0932 μg/g (hydrolyzed). With the scallop extracts the LOD values 0.0217μg/g (unhydrolyzed) and 0.0274 μg/g (hydrolyzed), and the LOQ values were 0.0372 μg/g (unhydrolyzed) and 0.0415 μg/g (hydrolyzed). The values in the hydrolyzed blanks were slightly higher than before hydrolysis but were still low enough to ensure detection and quantification of OA at the regulation level proposed by EU, 0.16μg/g whole meat [4]. The two limits were slightly higher with hydrolyzed samples, which might be due to the hydrolysis of a small amount of ester toxins contaminated in DG. Thus, the assay can detect and quantify OA in a very lower level than that of the regulation level proposed by EU (0.16 μg/g whole meat).

**Table 2:** Limits of detection and quantitation for shellfish.

**Matrix**	**Mussels**		**Scallops**		**Mean LOD **(μg/g DG)	**Mean LOQ** (μg/g DG)
	**Unhydrolyzed**	**Hydrolyzed**	**Unhydrolyzed**	**Hydrolyzed**		
Mean^a^(μg/g DG)	0.0295	0.0282	0.0152	0.0215		
SD	±0.0043	±0.0065	±0.0022	±0.002		
LOD^b^(μg/g DG)	0.0424	0.0476	0.0217	0.0274	0.0348	
LOQ^c^(μg/g DG)	0.0725	0.0932	0.0372	0.0415		0.0611
^a^ Mean value of 6 Independent blank shellfish samples; ^b^ Mean blank value x 3 SD; ^c^ Mean blank value x 10 SD.						

**Table 3:** Recovery of okadaic acid by the PP2A assay.

**Sample**	**OA concentration (μg/g DG)**		**SD**	**RSD**	**Recovery**
	**Spiked level**	**Mean**			
		(Detected by PP2A assay)		(%)	(%)
Mussels	0.1	0.0996	±0.0045	4.5	99.6
	0.2	0.1848	±0.0044	2.4	92.4
	0.3	0.2844	±0.0090	3.2	94.8
	0.4	0.4129	±0.0103	2.5	103.2
	0.5	0.4673	±0.0082	1.7	93.5
	1.0	1.0636	±0.0183	1.7	106.4
Scallops	0.1	0.0708	±0.0050	7.1	70.8
	0.2	0.1735	±0.0030	1.7	86.8
	0.3	0.2890	±0.0032	1.1	96.3
	0.4	0.3724	±0.0020	0.5	93.1
	0.5	0.4735	±0.0021	0.4	94.7
	1.0	1.0108	±0.0108	1.1	101.1
All assays were performed in triplicate.					

To evaluate the accuracy of the method, the shellfish DGs of mussels and scallops were spiked with known six different amounts (0.1, 0.2, 0.3, 0.4, 0.5, and 1.0 μg/g DG) of standard OA. The recovery of OA in mussels and scallops was 92.4-106% and 70.8-101%, respectively (Table 3). The low recovery (70.8%) scored in scallops spiked at a low dose (0.1μg) suggested an interfering substance.

**Figure 4:**
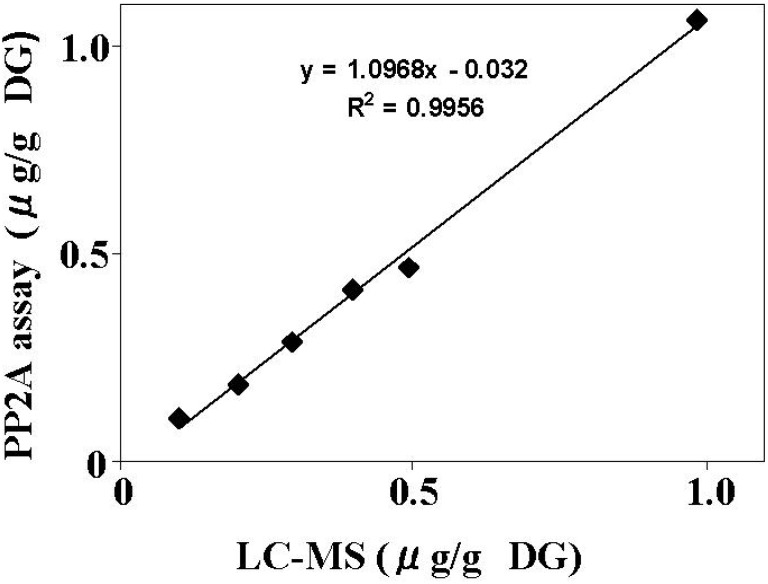
Comparison of the PP2A inhibition assay and LC-MS analysis. The mussel DG was spiked with OA at concentrations of 0.1, 0.2, 0.3, 0.4, 0.5 and 1 μg/g, and the extracts were subjected to the PP2A inhibition assay and LC/MS analysis. Each extract was measured in triplicate and the average value was compared between methods.

To evaluate the reproducibility of the assay method, six separate extractions were performed on each of the DG samples of mussels and scallops spiked at three levels of OA (0.1, 0.2, and 0.4 μg/g DG). Each extract was evaluated by the PP2A assay in six separate experiments. The RSD from the six separate experiments in mussels and scallops was 4.8-6.5% and 5.1-7.1%, respectively (Table 4). The result demonstrates high reproducibility of the PP2A assay. Furthermore, DG of mussels were spiked with OA (0.1, 0.2, 0.3, 0.4, 0.5, and 1.0 μg/g), and each extract was analyzed with the PP2A inhibition assay and the LC/MS method. As shown in Figure 4, a good correlation was observed between the two methods (R^2^ = 0.996) with the regression slope of 1.097. These data confirmed the reliability of the PP2A assay using rhPP2Ac as a method for detecting and quantifying OAs in shellfish.

**Table 4:** Reproducibility of data obtained by PP2A assay.

**Sample**	**OA concentration (μg/g DG)**		**SD**	**RSD** (%)	**Replicates**
	**Spiked level**	**Mean** (Detected by PP2A assay)			
Mussels	0.1	0.1019	±0.0066	6.5	6
	0.2	0.1898	±0.0121	6.4	6
	0.4	0.4079	±0.0194	4.8	6
Scallops	0.1	0.0711	±0.0041	5.8	6
	0.2	0.1713	±0.0122	7.1	6
	0.4	0.3596	±0.0184	5.1	6
All assays were performed in triplicate.					

## 3. Discussion

The development of rapid, sensitive, and inexpensive methods to monitor the DSP toxins occurrence in shellfish urgently is needed to manage the health risk posed by the toxins. Because these toxins inhibit PP2A strongly and specifically, PP2A is used to assay the DSP toxins. PP2A is one of the four major classes of eukaryotic serine/threonine phosphoprotein phosphatases [11,18]. The phosphatase activity is inhibited by several natural toxins, including OA [9,10] and microcystins [19]. Based on the specific inhibitory action, the PP2A inhibition assay method was proposed to determine OAs using *p*NPP [13] and was performed using enzymes purified from human red blood cells [15]. However, the assay method employing native PP2A have not been widely used because of fluctuations in enzyme quality. Since high purity, stability, and sensitivity are crucial for an enzyme to be used in microplate assays, we produced biologically active and highly pure rhPP2Ac from High Five insect cells using baculovirus expression system [16]. The advantage of using the recombinant PP2A for detection of the DSP toxins is the provision of the highly purified PP2A from the stable resource. In this study, we assessed the suitability of the rhPP2Ac on the PP2A inhibition assay for detection of OAs in shellfish.

Under our experimental condition, OA, DTX1, and hydrolyzate of 7-*O*-palmitoyl-OA dose-dependently inhibited rhPP2Ac at very low concentrations, ranging from 0.001 to 5 nM; the IC_50_ values of these DSP toxins were 0.095 ± 0.007 nM, 0.104 ± 0.006 nM, and 0.135 ± 0.009 nM, respectively (Figure 1 and Table 1), although PTX and YTX did not inhibit the activity of rhPP2Ac at comparable concentrations (Figure 2 and Table 1). Our results show that the PP2A assay using rhPP2A is more sensitive than that of using native PP2A, because the IC_50_ value of OA inhibiting the rhPP2Ac was lower than that of the purified PP2A from human red blood cells described previously [15,20]. These results indicate that the rhPP2Ac we produced can detect and quantify very low levels of OAs in shellfish and that interference from PTX and YTX will be of little importance. The high sensitivity of this assay indicates that rhPP2Ac is an excellent tool for constructing inhibition assay kits for DSP toxins.

A stable calibration curve was obtained with OA ranging from 0 to 10 ng/mL (Figure 3). The LOD and LOQ show that the assay is sensitive to meet the EU regulation level (0.16 μg/g whole meat) of OA (Table 2). Average recoveries of OA spiked into DG were more than 90%, and the RSD of the reproducibility was less than 7.1%. The good correlation observed between the PP2A inhibition assay and LC/MS data confirms the validity of the assay method. In conclusion, the PP2A inhibition assay, including that of hydrolysis, is a simple, sensitive, specific, accurate and reproducible method for the measurement of OA in shellfish. 

In this study, we chose the DG of a bivalve for extracting the toxins to perform the validation study, because the DSP toxins are known to localize in this organ. We will continue to accumulate enough data to validate assay performance for whole meat samples and other shellfish samples. The details of the validation study for the PP2A assay will be reported elsewhere. Nevertheless, the results obtained in the present study confirm that the rhPP2Ac we prepared is an excellent tool for detecting and quantifying OAs in shellfish. Therefore, the assay method employing the rhPP2Ac will be widely used for detecting OA.

## 4. Experimental Section

### 4.1. Lipophilic Toxins and Substrate Reagents and Solvents

OA was purchased from Wako (Osaka, Japan). DTX1, YTX and PTX1 were purified as reported previously [21]. 7-*O*-Palmitoyl-OA (pal-OA) was synthesized from OA according to the previous method [22]. The substrate used for the PP2A inhibition assay, *p*-nitrophenylphosphate (*p*-NPP), was purchased from Sigma (MO, USA). 

### 4.2. Purification of rhPP2Ac and Phosphatase Activity Assay

rhPP2Ac was synthesized in insect cells (High Five; Invitrogen, Carlsbad, CA, USA) by infection of recombinant baculoviruses encoding His_×__8_-tagged human PP2Acα using a baculovirus expression system and purified as described previously [16]. The purity of the recombinant human PP2Ac was confirmed by 12% sodium dodecyl sulfate-polyacrylamide gel electrophoresis (SDS-PAGE) using Coomassie brilliant blue R staining for visualization. The phosphatase activity assay using *p*-NPP as the substrate was performed as described previously [16]. One activity unit was defined as the amount of enzyme to release 1 nmol of phosphate from *p*-NPP per minute at 30 °C. 

### 4.3. PP2A Inhibition Assay

The experimental condition for the PP2A assay basically followed those of the previous method, except for the use of rhPP2Ac instead of a native dimeric enzyme [15]. The assay was carried out on 96-well plates. Each well contained 50 µl of lipophilic toxin and 100 µl of *p*-NPP (final concentration, 7.6 mM *p*-NPP per well). The reaction was started by adding 100 µl of the enzyme (final concentration, 0.08 units / well) and was continued for 30 min at 36 °C. The hydrolysis of *p*-NPP to *p*-NP was recorded on a microplate reader at 405 nm against 492 nm as a reference. The measurements were done in triplicate.

### 4.4. Preparation of Shellfish Samples for the PP2A Assay

The scallops (*Patinopecten yessoensis*) and blue mussels (*Mytilus galloprovincialis*) were purchased from local fish markets in Hokkaido and Aichi Prefecture in Japan. 

The digestive glands (DG) were separated from other tissues, minced, and a 2-g portion was used for extraction by Polytron homogenizer in 18mL of 90% methanol at room temperature for 1 min. The homogenate was centrifuged at 2,500g for 10 min, and a 50 μL of the supernatant (extract) was diluted with 950 μL of the sample dilution buffer to make a test solution. A 50-μL portion of the test solution was used for assaying of unhydrolyzed samples.

For hydrolysis and neutralization, the extract (500 μL) was mixed with 100 μL of 1.25N NaOH in a 2-mL plastic tube equipped with a screw cap. The cap was tightened and the tube was heated for 40 min at 80 °C, using a heating block. The reaction solution was cooled down to room temperature and neutralized by adding 100 μL of l.25N HCl. A 50 μL portion of the neutralized solution was diluted with 950 μL of the sample dilution buffer, and used for the PP2A inhibition assay. The OA content in the hydrolyzed samples was calculated by using a multiplying factor of 1.4 to calibrate the volume increase resulted by addition of alkali and acid solutions.

### 4.5. Assay Procedure

The PP2A inhibition assay was carried out on 96-well microplates. Each well was filled with 50 μL of the sample dilution buffer (blank), OA standard solution, or a test solution of shellfish extract. Then, 100 μL of the substrate solution (19mM *p*-NPP) and 100 μL of the enzyme solution (0.65 unit of PP2A) were added to each well. The microplate was sealed with an adhesive film, mixed for 1 min with a microplate mixer, and incubated for 30 min at 36 °C. The color development due to hydrolysis of *p*-NPP to *p*NP (*p*-nitrophenol) was measured with a microplate reader at 405 nm against 490 nm as reference within 10 min from the end of incubation. OA concentrations in shellfish extracts were determined by interpolating the absorbance values of the samples from the linear portion of the standard curve.

### 4.6. LC-MS Determination

LC-MS analysis was performed on a model 1100 liquid chromatograph (Agilent) coupled to a 4000Qtrap mass spectrometer (Applied Biosystems/MDS Sciex). Separation was carried out on a Capcellpak C18 MGII column (3 μm, ϕ2.1 x 100 mm, Shiseido, Japan) using gradient elution at 40 °C. Eluent A was water and B was acetonitrile- methanol (8:2, v/v), both containing 2 mM ammonium formate and 50 mM formic acid. Gradient elution from 40% to 100% B was performed over 20 min and then held at 100% B for 20 min for LC separation. The flow rate was 0.2 mL/min and the injection volume was 5 μL. Electrospray ionization in the negative mode was used. OA was monitored on the deprotonated [M-H]-ions at *m/z* 803 and the peak intensity was expressed in peak area. The OA content in sample extracts was quantified using a calibration curve prepared with reference OA. All samples and standards were injected three times in a continuous LC-MS run and the average response used.
